# Bidirectional association between knee osteoarthritis and depressive symptoms: evidence from a nationwide population-based cohort

**DOI:** 10.1186/s12891-022-05137-8

**Published:** 2022-03-05

**Authors:** Han Lu, Limin Wang, Weijiao Zhou, Shida Jin, Hongbo Chen, Yi Su, Nan Li, Shaomei Shang

**Affiliations:** 1 Peking University School of Nursing, No.38 Xueyuan Road, Haidian District, Beijing, China; 2 Zhejiang Chinese Medical University School of Nursing, No.548 Binwen Road, Binjiang District, Hangzhou, China; 3 University of Michigan School of Nursing, No.426 Ingalls Street, Ann Arbor, MI USA; 4 Peking University School of Public Health, No. 38 Xueyuan Road, Haidian District, Beijing, China; 5 Peking University Sixth Hospital, No.51 Hua Yuan Bei Road, Haidian District, Beijing, China; 6 Research Center of Clinical Epidemiology, Peking University Third Hospital, No.46 Hua Yuan Bei Road, Haidian District, Beijing, China

**Keywords:** knee osteoarthritis, depressive symptoms, longitudinal studies, proportional hazards models

## Abstract

**Background:**

Both knee osteoarthritis (KOA) and depressive symptoms (DS) are major public health issues affecting the quality of life. This study aimed to examine the association between KOA and DS.

**Methods:**

Data were gathered from the China Health and Retirement Longitudinal Study in 2011–2015 which surveyed middle-aged to elderly individuals and their spouses in 28 provinces in China. An adjusted Cox proportional hazards regression model was used to estimate hazard ratios (HRs).

**Results:**

The analysis for baseline KOA and the subsequent risk of DS was based on 2582 participants without baseline DS. During the follow-up, KOA patients were more likely to have DS than non-KOA participants (adjusted HR = 1.38: 95% CI = 1.23 to 1.83). The analysis for baseline DS and the subsequent risk of KOA was based on 4293 participants without baseline KOA, those with DS were more likely to develop KOA than non-DS participants (adjusted HR = 1.51: 95% CI = 1.26 to 1.81). Subgroup analysis showed sex and age had no significant moderating effect on the KOA-DS association.

**Conclusions:**

Our results provide evidence that the association between KOA and DS is bidirectional. Therefore, primary prevention and management of KOA and DS should consider this relationship.

**Supplementary Information:**

The online version contains supplementary material available at 10.1186/s12891-022-05137-8.

## Background

Knee osteoarthritis (KOA) and depressive symptoms (DS) are major physical health and mental health problems affecting the quality of life of older people, respectively. Specifically, KOA is one of the leading causes of disability in the elderly [1], accounting for approximately 85% of the burden of osteoarthritis (OA) worldwide [2]. Persons with KOA commonly experience pain, aching, stiffness, and associated functional loss [3]. Given the increased life expectancy and ageing of the global population, the prevalence, incidence, and years lived with disability due to KOA are expected to continue increasing in most countries [4]. What’s more, there is no known cure for KOA [5]. Depressive disorders ranked 13th in the causes of global disability-adjusted life-years (DALYs) in the latest Global Burden of Disease Study [6]. Multiple studies have found the aggregation of DS in KOA patients [7–11]. Combined depression/DS could aggravate patients’ pain and disability, reduce treatment adherence, and increase the healthcare burden [12–14]. Agarwal P et al. found that OA patients with depression could increase the annual medical expenses by nearly US$4400 [15]. Therefore, it is necessary to pay more attention to the KOA with depression/DS as a major public health issue.

There are two possible explanations for the aggregation of DS in KOA patients: patients with KOA have a high risk to develop depression, or depressed persons are more likely to develop KOA. This has not been determined yet. On the one hand, DS is reportedly highly prevalent among KOA patients, and KOA has been cited as a risk factor for developing DS [7–11]. On the other hand, some studies have shown that patients with DS are more likely to develop arthritis [11, 16]. Although many studies have explored the single-directional relationship between KOA and DS, whether the relationship between KOA and DS in both directions coexists in the same group is less verified. Moreover, most of the current studies were based on population in developed countries, and there is a lack of evidence from China.

To fill this gap, our study aimed to use data from a middle-aged and elderly cohort to determine whether the association between KOA and DS is bidirectional and the strength of association. Firstly, we analyzed the effect of baseline KOA on the incidence of subsequent DS; secondly, we analyzed the effect of baseline DS on the incidence of subsequent KOA. Besides, we explore the strength of these effects at different age, sex groups.

## Methods

### Participants

Data for this study were gathered from the baseline survey in 2011 and two follow-up surveys in 2013 and 2015 of the China Health and Retirement Longitudinal Study (CHARLS). The CHARLS national survey used a multistage sampling strategy covering 28 provinces, 150 counties or districts, and 450 villages or urban communities in China. Successfully interviewed 17,708 individuals from 10,257 households, representing the middle-aged and elderly people in China in general [17]. In the first analysis to predict the incidence of DS, participants were eligible if (i) they were ≥ 45 years old; (ii) they did not have DS at baseline; (iii) they were followed up for ≥1 year, and status of DS during follow-up was recorded; (iv) they had complete baseline information (including KOA status and covariates). The second analysis to predict the incidence of KOA included participants if (i) they were ≥ 45 years old; (ii) they did not have KOA at baseline; (iii) they were followed up for ≥1 year, and status of KOA during follow-up was recorded; (iv) they had complete baseline information (including DS status and covariates).

### Measures

#### KOA onset

All participants had a face-to-face household interview using a structured questionnaire. According to the definition in a previous study [18], KOA was identified by self-reported both physician-diagnosed osteoarthritis and the presence of concurrent knee pain. Physicians who made the diagnosis include internists, rheumatologists, orthopedic surgeons and doctors in Chinese medicine, and the diagnosis is often reached with the aid of X-rays [18, 19].

#### Depressive symptoms

DS was measured by the Chinese version of the Center for Epidemiologic Studies Depression (CES-D), a 10-item questionnaire with good reliability and validity (possible range, 0 ~ 30) [20]. The presence of DS was defined by a CES-D score of 10 or higher [20].

#### Covariates

Covariates in the analysis included sociodemographic characteristics, lifestyle, and health-related factors at baseline. All the variables were obtained from the 2011 CHARLS survey. As described in previous studies [8, 21, 22], the following factors were selected as covariates: a) sociodemographic characteristics: age, sex, marital status, education level, yearly income [23], and residence (urban, rural); b) lifestyle: smoking status, drinking status, length of sleep, physical activity (calculated using metabolic equivalent multiplied by activity weekly duration level) [24, 25]; c) health-related factors: whole health and childhood health status (a 5-point Likert scale, where higher scores mean better health), body mass index (BMI), the number of difficulties with activities of daily living (ADL) [26] and instrumental activities of daily living (IADL) [27]. the number of non-communicable diseases (NCDs), experienced major accidental injury or not, and C-reactive protein (CRP) [21]. Except for BMI and CRP were measured on site, all variables were obtained from self-reported questionnaire data, and the detailed information related to how each covariate was assessed and the used tools is presented in Supplementary Table S1.

### Statistical analysis

Continuous variables are reported as mean ± standard deviation (SD), while categorical variables are expressed using proportions. We used t-tests and chi-square tests to analyze differences among groups. We used Kaplan–Meier plots to show the incidence of DS by KOA or non-KOA, and the incidence of KOA by DS or not. Log-rank tests were used to compare the differences between groups. The duration of follow-up was defined as the date of the baseline interview to either the date of DS/KOA onset or the date of the last interview. The incidence density of KOA and DS was estimated by the number of incident patients divided by the number of person-years accumulated in the population. The proportional hazards assumption was checked using the Schoenfeld residuals test. As the test results were reported as *P* > 0.05, the proportional hazard assumptions were satisfied. Cox proportional-hazard regression was used to calculate the crude and adjusted hazard ratios (HRs) with corresponding 95% confidence intervals (CIs). Furthermore, we performed stratified Cox regression analysis to examine the modifying effect of age (< 65 years and ≥ 65 years), sex on the bidirectional KOA–DS associations. All tests were 2-tailed, and the significance level was set to *P* < 0.05. Stata 15.1 was used for analysis.

To check whether the analyzed sample was representative of the entire population, we described the characteristics of the CHARLS population and the middle-aged and elderly KOA and DS incident cohorts, as well as the distribution of missing values (Supplementary Table S2). Considering the potential bias caused by excluding missing data participants, we performed sensitivity analyses comparing crude HRs between the analytical sample and the incident cohorts with missing values (Supplementary Table S3 and S4).

## Results

### The cohort analysis for baseline knee osteoarthritis and the subsequent risk of depressive symptoms

**Fig. 1:**
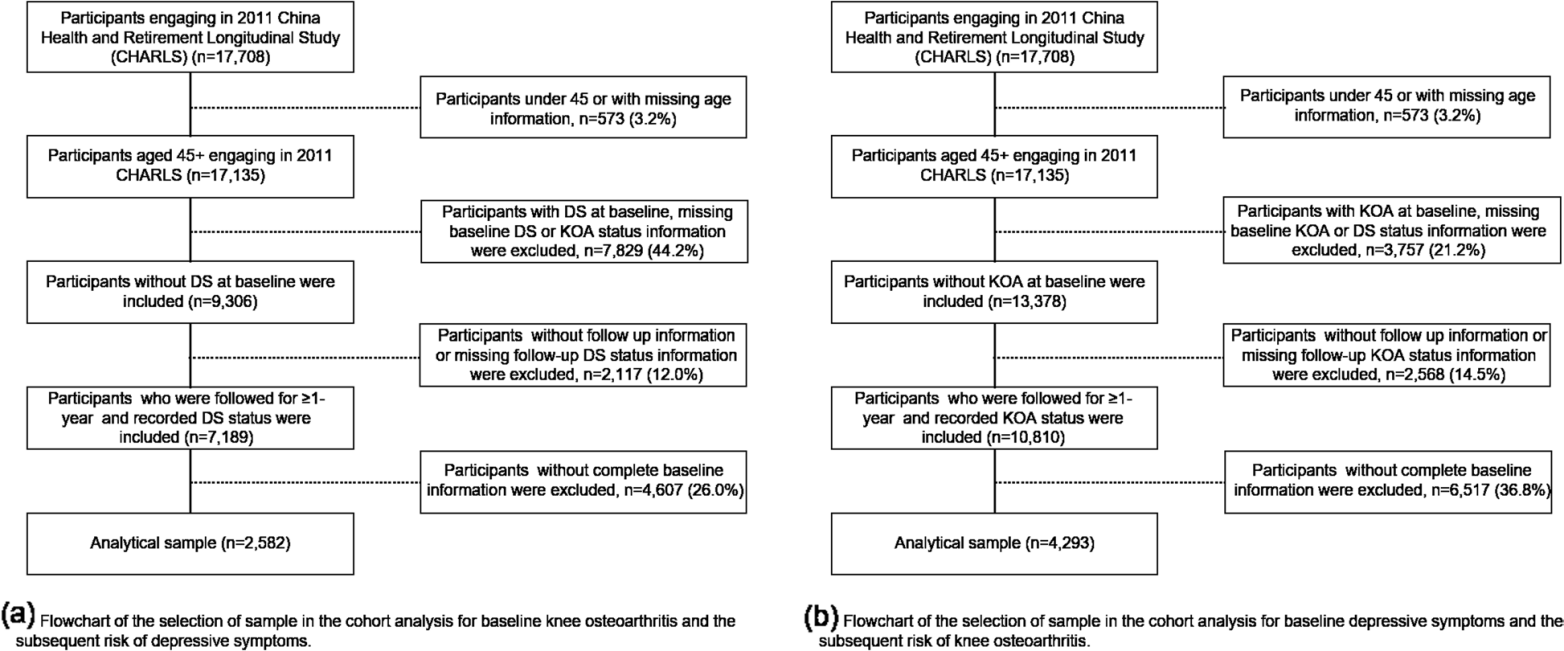
Flowchart of the selection of sample

The analysis for the risk of DS with KOA was based on 2582 participants free from DS at baseline (See Fig. 1 for the selection process). Table 1 shows the baseline characteristics of the KOA and non-KOA participants. KOA was found more frequently among women, rural residents, those with at most primary education and low-income persons. Other characteristics associated with KOA were fewer sleep hours, worse whole health, worse childhood health, higher BMI, more difficulties with ADL and IADL, more NCDs, and more accidental injury (*P* < 0.05 for all these variables).

**Table 1:** Baseline Characteristics of Participants Included in the Analysis

		Baseline KOA and the Subsequent Risk of DS			Baseline DS and the Subsequent Risk of KOA		
		Without KOA at baseline	With KOA at baseline	*P*	Without DS at baseline	With DS at baseline	*P*
		(*n* = 2402)	(*n* = 180)		(*n* = 2763)	(*n* = 1530)	
Sex	Male (%)	1377 (57.33)	88 (48.89)	0.028	1571 (56.86)	675 (44.12)	< 0.001
	Female (%)	1025 (42.67)	92 (51.11)		1192 (43.14)	855 (55.88)	
Age [mean (SD), years]		60.00 (0.20)	60.55 (0.70)	0.46	59.31 (0.18)	60.12 (0.25)	0.008
Education	Primary school and below (%)	1507 (62.74)	134 (74.44)	0.003	1748 (63.26)	1181 (77.19)	< 0.001
	Middle/high/vocational school (%)	847 (35.26)	46 (25.56)		962 (34.82)	341 (22.29)	
	Associate degree and above (%)	48 (2.00)	0 (0.00)		53 (1.92)	8 (0.52)	
Marital status	Unmarried (%)	392 (16.32)	25 (13.89)	0.39	573 (20.47)	472 (30.85)	< 0.001
	Married (%)	2010 (83.68)	155 (86.11)		2190 (79.26)	1058 (69.15)	
Yearly income	Under average level (%)	1346 (56.04)	120 (66.67)	0.005	1522 (56.17)	980 (64.05)	< 0.001
	Average and above (%)	1056 (43.96)	60 (33.33)		1211 (43.83)	550 (35.95)	
Residence	Rural (%)	1919 (79.89)	157 (87.22)	0.017	2229 (80.67)	1337 (87.39)	< 0.001
	Urban (%)	483 (20.11)	23 (12.78)		534 (19.33)	193 (12.61)	
Smoke	Current smoker (%)	852 (35.47)	61 (33.89)	0.83	978 (35.40)	451 (29.48)	< 0.001
	Previous smoker (%)	244 (10.16)	17 (9.44)		283 (10.24)	140 (9.15)	
	Never smoked (%)	1306 (54.37)	102 (56.67)		1502 (54.36)	939 (61.37)	
Drink	No (%)	1477 (61.49)	117 (65.00)	0.35	1699 (61.49)	1102 (72.03)	< 0.001
	Yes (%)	925 (38.51)	63 (35.00)		1064 (38.51)	428 (27.97)	
Length of sleep [mean (SD), hours]		6.71 (0.03)	6.31 (0.15)	0.003	6.71 (0.03)	5.85 (0.05)	< 0.001
Physical activity (SD)		60.31 (2.09)	71.28 (8.59)	0.17	62.94 (2.00)	61.52 (2.75)	0.68
Whole health status score (SD)		3.31 (0.02)	2.67 (0.05)	< 0.001	3.25 (0.02)	3.84 (0.02)	< 0.001
Childhood health status score (SD)		3.34 (0.02)	3.13 (0.09)	0.011	3.33 (0.02)	3.16 (0.03)	< 0.001
BMI	< 18.5 kg / m2 (%)	132 (5.50)	10 (5.56)	0.025	145 (5.25)	151 (9.87)	< 0.001
	18.5–23.9 kg / m2 (%)	1266 (52.71)	76 (42.22)		1443 (52.23)	847 (55.36)	
	24–27.9 kg / m2 (%)	718 (29.89)	62 (34.44)		842 (30.47)	386 (25.23)	
	≥28 kg / m2 (%)	286 (11.91)	32 (17.78)		333 (12.05)	146 (9.54)	
Number of difficulties in ADL (SD)		1.23 (0.04)	3.56 (0.19)	< 0.001	1.40 (0.04)	2.98 (0.07)	< 0.001
Number of difficulties in IADL (SD)		0.16 (0.01)	0.47 (0.07)	< 0.001	0.17 (0.01)	0.58 (0.03)	< 0.001
Number of non-communicable diseases (SD)		1.02 (0.02)	2.63 (0.11)	< 0.001	1.12 (0.02)	1.60 (0.04)	< 0.001
With major accidental injury (%)		185 (7.70)	28 (15.56)	< 0.001	225 (8.14)	171 (11.18)	0.001
CRP	≤3 mg/l (%)	1976 (82.26)	144 (80.00)	0.45	2263 (81.90)	1233 (80.59)	0.29
	> 3 mg/l (%)	426 (17.74)	36 (20.00)		500 (18.10)	297 (19.41)	

*BMI* body mass index, *ADL* activities of daily living, *IADL* instrumental activities of daily living, *CRP* C-reactive protein

**Fig. 2:**
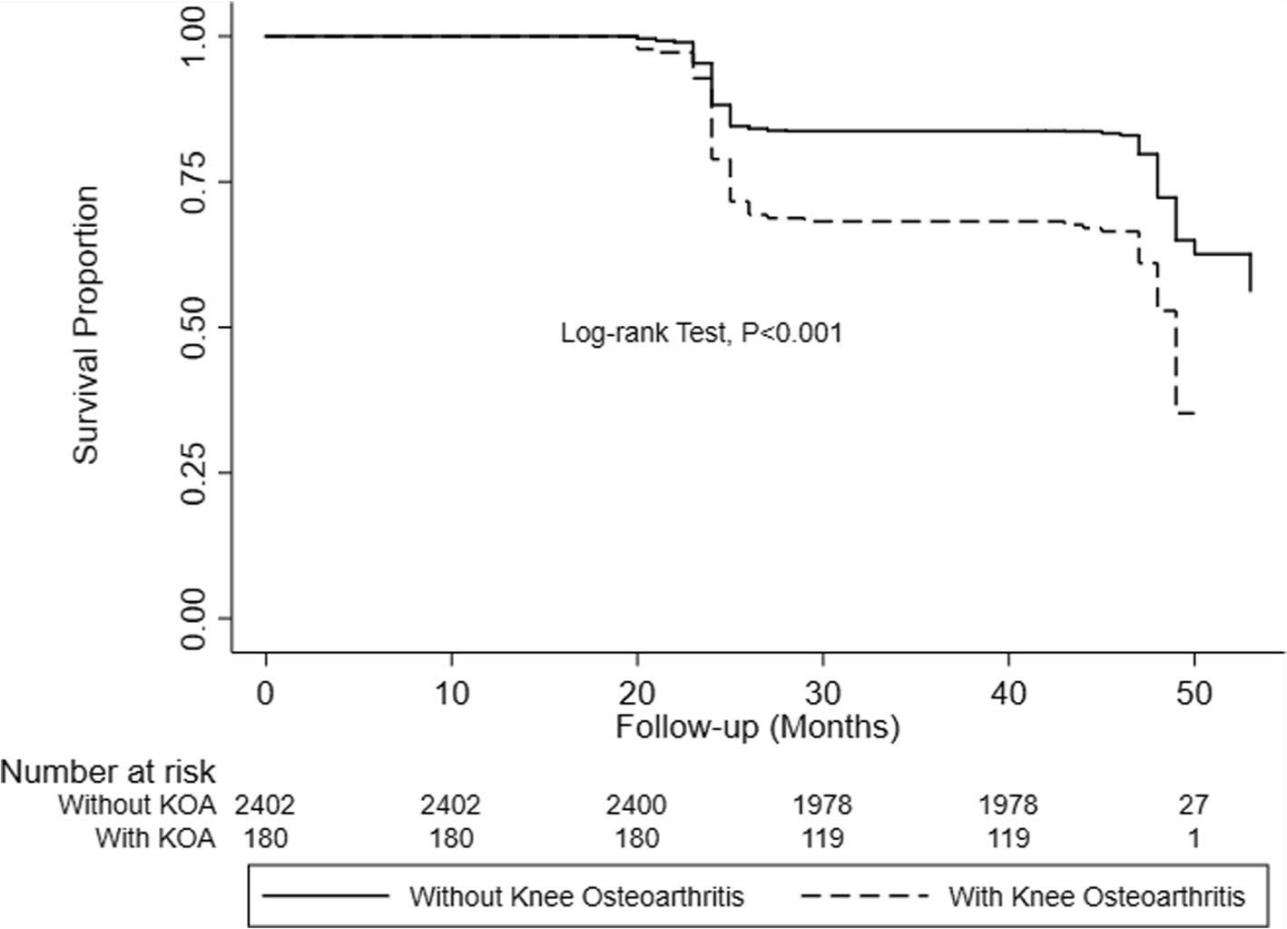
The proportion of patients remaining free from depressive symptoms during four years follow-up

During the four-year follow-up, 756 individuals (29.3% of the baseline population) developed DS, with an incidence of 8.22 new cases per 1000 person-months. Specifically, the incidence density of DS in KOA patients was 15.48 per 1000 person-months, compared with 7.73 per 1000 person-months among non-KOA participants (Table 2). The Kaplan–Meier survival analysis showed that participants with KOA had an increased risk of DS (Fig. 2). Cox proportional hazards regressions further demonstrated the effect of KOA on DS. Participants with KOA were more likely to develop DS than those without KOA (HR 2.05; 95% CI = 1.65 to 2.56). After adjusting for all potential confounders, this effect was still statistically significant. Among all subgroups, persons with KOA had a higher incidence of DS, with females < 65 having the highest incidence (Table 2). The stratified analysis found that age and sex did not modify the association between baseline KOA and incident DS, the *P*-value for KOA-sex interaction and KOA-age interaction was 0.993 and 0.474, respectively.

**Table 2:** Incidence and Hazard Ratios for Depressive Symptoms in Association with Knee Osteoarthritis, Overall and Stratified by Age and Sex (*N* = 2582)

	With KOA at baseline		Without KOA at baseline		Adjusted HR* (95% CI)	***P***
	Event/at risk (n / person-months)	Incidence rate per 1000 person-months	Event/at risk (n / person-months)	Incidence rate per 1000 person-months		
**Overall**	90 / 5814	15.48	666 / 86,164	7.73	1.38 (1.07–1.78)	< 0.001
**Age < 65**	64 / 4155	15.40	481 / 63,737	7.55	1.53 (1.13–2.07)	0.007
Male	23 / 2075	11.08	218 / 36,283	6.01	1.5 (0.91–2.46)	0.11
Female	41 / 2080	19.71	263 / 27,454	9.58	1.63 (1.10–2.42)	0.015
**Age ≥ 65**	26 / 1659	15.67	185 / 22,427	8.25	1.29 (0.81–2.06)	0.29
Male	13 / 939	13.84	101 / 14,641	6.90	1.3 (0.65–2.58)	0.46
Female	13 / 720	18.06	84 / 7786	10.79	1.1 (0.54–2.22)	0.79

The *P*-value for KOA-sex interaction was 0.993. The *P*-value for KOA-age interaction was 0.474.

### The cohort analysis for baseline depressive symptoms and the subsequent risk of knee osteoarthritis

The parallel analysis of DS predicting KOA onset was based on 4293 participants free from KOA at baseline (see Fig. 1 for the selection process and Table 1 for baseline characteristics). Compared with those without DS at baseline, participants with DS had a higher proportion of females, aged 65 and older, less educated, unmarried, low-income population, and rural residents. On the other hand, there were fewer smokers and drinkers among patients with DS, and their health status was worse (embodied in the length of sleep, overall health, childhood health, BMI index, difficulties with ADL and IADL, number of NCDs, and injury events).

**Fig. 3:**
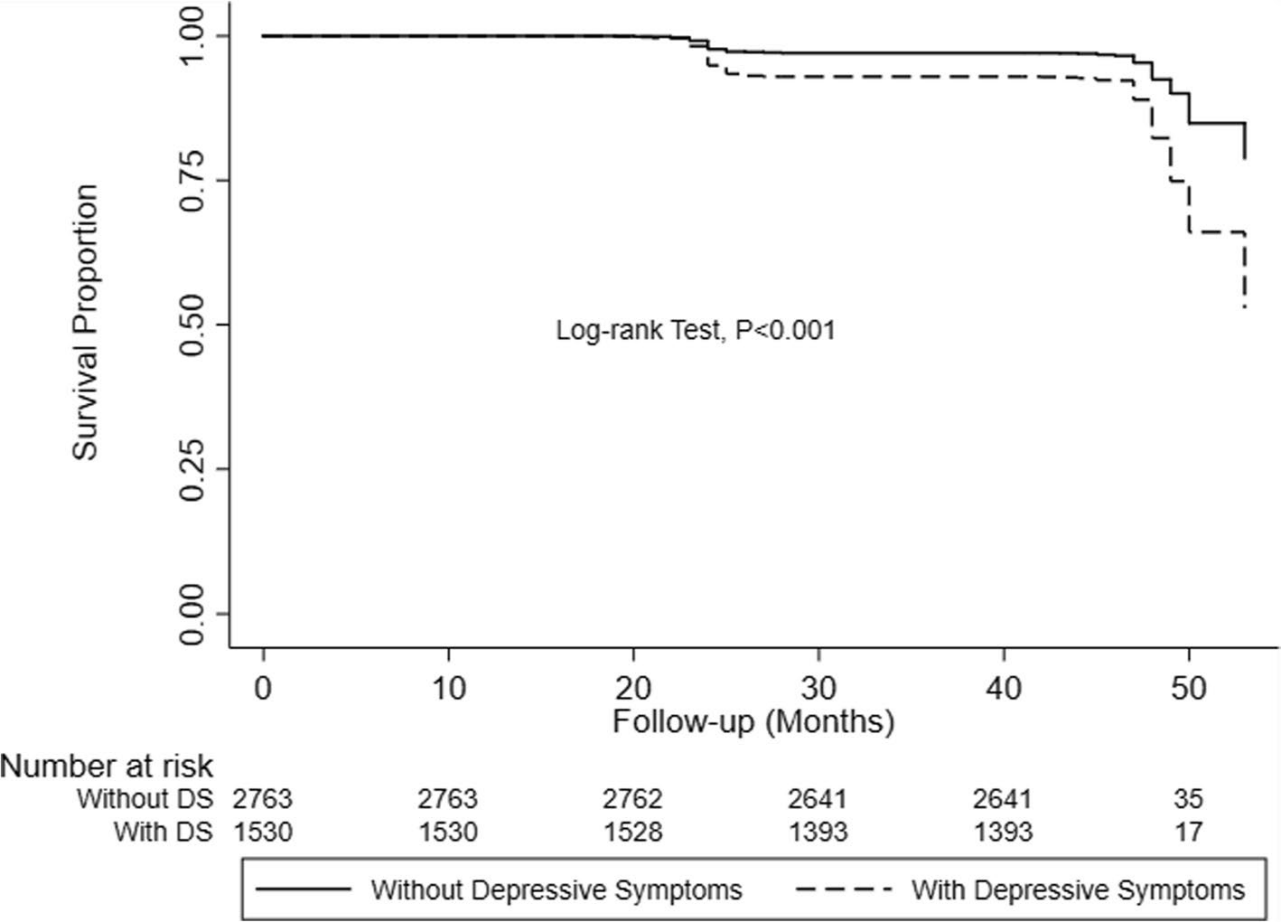
The proportion of patients remaining free from knee osteoarthritis during four years follow-up

Over the four years, 469 individuals (10.9%) developed KOA, with an incidence of 2.40 new cases per 1000 person-months. In the group of individuals with DS, the incidence density of KOA was 3.89 per 1000 person-months, which was 2.45 times that of the control group (Table 3). Kaplan–Meier survival analysis and Cox proportional hazards regression suggested that patients with DS had a higher risk of developing KOA (Fig. 3); crude HR for KOA associated with baseline DS was 2.52 (95% CI = 2.10 to 3.03), and the adjusted HR was 1.51 (95% CI = 1.23 to 1.84) (Table 3). In the subgroup analysis, regardless of age and sex, the incidence of KOA in patients with baseline DS was higher than in patients without baseline DS, with females aged < 65 years having the highest incidence rate (Table 3). DS-sex interaction and DS-age interaction analysis showed that neither age nor sex mediated the association between DS and KOA.

**Table 3:** Incidence and Hazard Ratios for Knee Osteoarthritis in Association with Depressive Symptoms, Overall and Stratified by Age and Sex (*N* = 4293)

	With DS at baseline		Without DS at baseline		Adjusted HR (95% CI)	***P***
	Event / at risk	Incidence rate	Event/at risk	Incidence rate		
	(n / person-months)	per 1000 person-months	(n / person-months)	per 1000 person-months		
**Overall**	267 / 68,565	3.89	202 / 126,723	1.59	1.51 (1.23–1.84)	< 0.001
**Age < 65**	192 / 48,068	3.99	140 / 91,970	1.52	1.62 (1.27–2.07)	< 0.001
Male	68 / 19,965	3.41	58 / 50,866	1.14	1.64 (1.09–2.48)	0.017
Female	124 / 28,103	4.41	82 / 41,104	1.99	1.59 (1.16–2.17)	0.004
**Age ≥ 65**	75 / 20,497	3.66	62 / 34,753	1.78	1.35 (0.93–1.96)	0.115
Male	34 / 10,397	3.27	27 / 21,430	1.26	1.34 (0.74–2.40)	0.33
Female	41 / 10,100	4.06	35 / 13,323	2.63	1.31 (0.79–2.16)	0.29

The *P*-value for DS-sex interaction was 0.346. The *P*-value for DS-age interaction was 0.325.

## Discussion

This national cohort analysis provided compelling evidence that the association between KOA and DS is bidirectional. We found that patients with baseline KOA had a higher incidence of DS and their risk of developing DS within four years was 1.38 times that of persons without baseline KOA. In contrast, a higher incidence of KOA can be identified in individuals with baseline DS. Baseline DS increased the risk of KOA by 51% over four years of follow-up. This two-way association was independent of sociodemographic factors and individual lifestyle and health-related factors.

Existing evidence has focused mainly on the risk of depression in KOA patients. A recent meta-analysis demonstrated that approximately 18.5% of KOA patients also have DS, which is higher than the prevalence in the general population [9, 28]. Nicola et al. reported that the incidence of DS associated with KOA was 26 per 1000 person-years, KOA patients were more likely to develop DS (odds ratio, = 1.43: 95% CI = 1.03 to 1.98) [29]. Another study analyzing the association of cancer, cardiovascular disease, diabetes, and OA at baseline with incident depression found that among these four conditions OA had the highest risk for incident depression (HR = 1.94: 95% CI = 1.80 to 2.10) [30]. However, most of the existing research samples have come from high-income countries, whereas our research provides evidence from China. And the incidence of DS related to KOA in China is higher than that reported in previous studies in other countries. There are several studies focusing on the risk of KOA in patients with DS. Previous analyses on the relationship between OA and depression showed similar results to ours, that is, the presence of DS could increase the risk of OA [11, 16].

The actual mechanisms contributing to the association between KOA and DS have not been well understood yet. Regarding the susceptibility of KOA patients to DS, depression and DS in patients with chronic diseases are usually attributed to the emotional response to the diagnosis of the disease, or the lifestyle limitations due to the disease [31]. Studies have suggested that pain, disability, reduced physical activity, obesity, and sleep disturbance might be important causes for DS among KOA patients [32, 33]. This study also found that longer sleep duration and better overall health status were important protective factors for developing DS (Supplementary Table S5). As for why depressed patients are prone to KOA, there is currently a lack of relevant research. According to previous studies, depressed individuals are more likely to adopt unhealthy lifestyles, such as unhealthy diets and sedentary behavior, which are risk factors for KOA [34, 35]. Milaneschi Y et al. found that compared with controls [36], patients with depression had more vitamin D deficiency, while evidence showed that low vitamin D level was an important predictor of KOA [37]. Concurrently, research implied that both KOA and depression could be regulated by similar inflammatory immune mechanisms. The production and release of TNFα, IL-1β, IL-6, and IL-8 have been detected in the synovial fluid, synovial tissue and serum of patients with KOA [38, 39]. These pro-inflammatory factors participate in the pathogenesis of KOA through the hypothalamic-pituitary-adrenal axis, causing crosstalk between the brain and the immune system. On the other hand, these pro-inflammatory factors are also important factors regulating central pain and depression. Donovan et al. have tested TNF-α, IL-6, IL-10, CRP, and other inflammatory factors in patients with depression and healthy controls and found that these indicators were independently related to patients’ suicidal ideation [40]. However, the CRP (≤3 mg/l or > 3 mg/l) included in this analysis was not observed to affect the incidence of KOA and DS (Supplementary Table S5 and S6). Further research considering biological, behavioral, and physiological pathways is needed to understand the mechanisms of this association.

In the subgroup analysis, we found that in all age and sex groups, participants with baseline KOA had a higher incidence of DS, accordingly, participants with baseline DS had a higher incidence of KOA. This result is consistent with our assumptions. As for some subgroups, the risk of DS associated with KOA and the risk of KOA associated with DS were not statistically significant, which may be mainly related to insufficient sample size. Similar to other surveys [3, 41], both KOA and DS are very common among middle-aged and older people, especially among females. However, gender was not identified as a mediator in the relationship between KOA and DS in this research, which is consistent with another longitudinal study [30]. And we found no mediating effect of age, either. A weakened relationship between KOA and DS was observed in the 65 + −year age group compared to the 45–65-year age group. One reason may be a selection bias in this study. Because people aged ≥65 years are more likely to suffer from long-term KOA or DS, it may be more common to find people with both DS and KOA among those aged ≥65 years. However, these people were excluded, and selected older participants may be less susceptible to the bidirectional relationship between KOA and DS. Beyond that, studies have also offered some explanations. The decreased risk of DS associated with KOA in the elderly group may be because older people pay less attention to the functional impairment caused by KOA than middle-aged people [42], while middle-aged were still juggling many roles (such as family and work responsibilities) and did not expect difficulties with their daily activities. Similarly, the HR for KOA associated with baseline DS was smaller in older adults than in middle-aged. Evidence showed that older patients with DS may increase the competing risk of death from other causes [43, 44], which masked the incidence of KOA. Also, a previous study revealed that older adults may less likely to report DS [45], and the non-differential misclassification of DS could bias the association towards null.

### Implications for mental health nursing

KOA and DS often coexist in patients, which leads to increased medical costs and a worse prognosis [9, 12, 15]. We need to pay attention to the coexistence of KOA and DS, and explore the association between the two. This study proved a bidirectional association between KOA and DS through cohort analysis, patients with KOA have more DS than the general population, and depressed patients are more likely to develop KOA.

Considering the susceptibility of KOA patients to DS, there is a need to incorporate depression screening, high-risk group prevention, and treatment into the management of KOA patients. The National Institute for Health and Clinical Excellence (NICE) also recommended that patients with OA should strengthen the treatment and management of depression [46]. When facing depression patients, osteoarthritis prevention guidance also needs to be carried out. Exercise therapy as an important non-pharmacological treatment has been found to not only bring specific KOA prevention benefits, but also improve quality of life and psychosocial factors [47, 48]. Previously, it has been found that there is a strong connection between coronary heart disease/chronic obstructive pulmonary disease/diabetes and depression/DS, the bidirectional association between KOA and DS once again proves the interactive relationship between physical health and mental health [49–51]. It is necessary to pay attention to the bidirectional association in the practice, and strengthen the communication and cooperation between mental health practitioners and physical health practitioners.

### Study strengths and limitations

The strengths of this study are the longitudinal study design and the use of population-based data that provide sufficient sample size and statistical power, as well as the validated and widely used 10-item CES-D. Nevertheless, this study had several limitations. First, we adopted self-reported physician-diagnosed osteoarthritis and knee pain to determine KOA, with possible chances for misclassification. Second, we analyzed only baseline KOA and baseline DS, that is, without considering any KOA or DS that might emerge during the follow-up period, which may reduce the apparent effects of KOA on the incidence of DS and of DS on the incidence of KOA. But this still does not prevent us from concluding there is a bidirectional relationship between KOA and DS. And there may be bias in the older subgroup analysis by excluding older adults with both KOA and DS at baseline. Third, we excluded participants without complete covariates information which may lead to selection bias, however, the sensitivity analysis showed only small differences of crude HRs between included sample and incident cohorts with missing values. Besides, the database does not contain information about the severity of KOA and treatments information (only includes the degree of pain, but it cannot be included in the analysis because most of the population lacks this data), so we cannot assess the relationship between the severity of KOA or treatments and subsequent DS risk.

## Conclusions

KOA and DS are major problems that plague the health of older adults. Patients with KOA often suffer from DS, which reduces their quality of life and increases the healthcare burden. This large, well-established cohort study provides compelling evidence that the association between KOA and DS is bidirectional. And this association cannot be fully explained by risk factors such as age, sex, BMI, physical activity, sleep time, and disabilities. Our findings remind both mental health practitioners and physical health practitioners to carefully consider this bidirectional association in the primary prevention and management of KOA and DS. When facing patients with KOA or depression/DS, it is crucial to strengthen the screening and treatment of another disease while treating the visiting disease.

## Supplementary Information

**Additional file 1.** Supplementary materials. Table S1-S6.

## Data Availability

All data used in this study are available in public, and the access policy and procedures are available at: http://charls.pku.edu.cn/index/en.html.
