# Supplementary material for: Bidirectional association between knee osteoarthritis and depressive symptoms: evidence from a nationwide population-based cohort
Source: BMC Musculoskelet Disord. 2022 Mar 5;23:213. doi: 10.1186/s12891-022-05137-8 (PMC8897877; doi:10.1186/s12891-022-05137-8)
Supplement: Supplementary file 1 — Additional file 1. Supplementary materials. Table S1-S6. [file 12891_2022_5137_MOESM1_ESM.docx]

**Supplementary Material**

**Table S1** Covariates and Methods of Measurement

| **Covariates** | **Methods of measurement** |
| --- | --- |
| Age | As a continuous variable in the main analyses, classified as 45-59 years and ≥60 years in the subgroup analyses |
| Sex | Male, female |
| Marital status | Married, unmarried |
| Education level | Primary school and below, middle/high/vocational school, associate degree and above |
| Yearly income | Lower than average per capita annual income of urban/rural residents, higher than average per capita annual income of urban/rural residents. Specifically, in 2011, the average annual income per capita in China was 6,977 yuan in rural areas and 23,979 yuan in urban areas (Statistics 2012) |
| Residence | Urban, rural |
| Smoking status | Current smoker, previous smoker, never smoked |
| Drinking status | Yes, no |
| Length of sleep | Measured by asking the question, “During the past month, how many hours of actual sleep did you get at night” |
| Physical activity | The number of days the participants spent in the past week on different types of physical activity (vigorous activity, moderate activity, and walking for at least 10 consecutive minutes) and time spent per day (time intervals and assigned index as follows:1 = < 0.5 h; 2 = 0.5–2 h; 3 = 2–4 h; and 4 = > 4 h) were collected in the “health status and functioning” section of CHARLS. Vigorous activities refer to the activities that make participants breathe much harder than normal and may include heavy lifting, digging, plowing, aerobics, fast bicycling, and cycling with a heavy load. Moderate activities refer to the activities that make participants breathe somewhat harder than normal and may include carrying light loads, bicycling at a regular pace, or mopping the floor. Walking includes at work and home, walking to travel from place to place, and any other walking that you might do solely for recreation, sport, exercise, or leisure.  Physical activity scores were calculated based on the scoring method of the International Physical Activity Questionnaire (IPAQ). Firstly, the weekly duration score was calculated by multiplying the number of days and the daily duration index for each activity. Subsequently, the intensity of physical activity refers to the metabolic equivalents (MET) of different physical activities specified by the IPAQ, MET levels for vigorous activity, moderate activity, and walking were 8.0, 4.0, and 3.3, respectively. Finally, physical activity scores = 8.0 × total vigorous activity weekly duration score + 4.0 × total moderate activity weekly duration score + 3.3 × total walking weekly duration score (Bai et al. 2021; Craig et al. 2003). |
| Whole health and childhood health status | Measured by asking a 5-point Likert scale problem “would you say your health is excellent, very good, good, fair, poor?” or “How would you evaluate your health during childhood, up to and including age 15? Excellent, very good, good, fair, poor?”, where higher scores mean better health |
| Body mass index (BMI) | Calculated as measured weight (kg) divided by height squared (m2), and categorized as underweight [< 18.5 kg/m2], normal [18.5–23.9 kg/m2], overweight [24.0–27.9 kg/m2], and obese [≥28.0 kg/m2] |
| The number of difficulties with activities of daily living (ADL) | Measured using the Katz ADL (Katz et al. 1963), including dressing, bathing, eating, transfer, continence, and toileting |
| The number of difficulties instrumental activities of daily living (IADL) | Measured using the Lawton IADL (Lawton et al. 1969), including doing housework, cooking, shopping, making phone calls, taking medication, and managing money |
| The number of non-communicable diseases | Measured based on self-reports of physician-diagnosed chronic diseases including hypertension, dyslipidemia, diabetes or high blood sugar, cancer, chronic lung diseases, liver disease, coronary heart disease, stroke, kidney disease, digestive disease, psychiatric problems, memory-related disease, arthritis, and asthma |
| With major accidental injury | Self-report to check whether one experienced a traffic accident or any other kind of major accidental injury or not |
| C-reactive protein (CRP) | Tested using venous blood samples and Immunoturbidimetric assay method with 3 mg/l as cutoff (Okely, Weiss, and Gale 2017) |

**References**

Bai, A., L. Tao, J. Huang, J. Tao, and J. Liu. 2021. 'Effects of physical activity on cognitive function among patients with diabetes in China: a nationally longitudinal study', *BMC Public Health*, 21: 481.

Craig, C. L., A. L. Marshall, M. Sjostrom, A. E. Bauman, M. L. Booth, B. E. Ainsworth, M. Pratt, U. Ekelund, A. Yngve, J. F. Sallis, and P. Oja. 2003. 'International physical activity questionnaire: 12-country reliability and validity', *Med Sci Sports Exerc*, 35: 1381-95.

Katz, S., A. B. Ford, R. W. Moskowitz, B. A. Jackson, and M. W. Jaffe. 1963. 'STUDIES OF ILLNESS IN THE AGED. THE INDEX OF ADL: A STANDARDIZED MEASURE OF BIOLOGICAL AND PSYCHOSOCIAL FUNCTION', *JAMA : the journal of the American Medical Association*, 185: 914-19.

Lawton, M. Powell, M. P. Lawton, Elaine M. Brody, and E. M. Brody. 1969. 'Assessment of older people: Self-maintaining and instrumental activities of daily living', *Gerontologist*, 9: 179-86.

Okely, J. A., A. Weiss, and C. R. Gale. 2017. 'Well-Being and Arthritis Incidence: The Role of Inflammatory Mechanisms. Findings From the English Longitudinal Study of Ageing', *Psychosom Med*, 79: 742-48.

National Bureau of Statistics. 2012. 'Statistics Bureau announced: National urban and rural residents' income growth in 2011 in China.', Accessed Oct15. <http://www.gov.cn/gzdt/2012-01/20/content_2050056.htm>.

**Table S2** Baseline Characteristics and Missing Value Distribution of Total CHARLS Participants, Incident Depressive Symptoms (DS) Cohort with Missing Values for Covariates, and Incident Knee Osteoarthritis (KOA) Cohort with Missing Values for Covariates

|  |  | **Total CHARLS participants  (n=17,808)** | | **Incident DS cohort with missing values for covariates  (n=7,189)** | | **Incident KOA cohort with missing values for covariates  (n=10,810)** | |
| --- | --- | --- | --- | --- | --- | --- | --- |
|  |  | n (%) | missing values | n (%) | missing values | n (%) | missing values |
| Gender | Male | 8471 (47.88) | <1% | 3963 (55.17) | <1% | 5368 (49.69) | <1% |
|  | Female | 9221 (52.12) |  | 3220 (44.83) |  | 5436 (50.31) |  |
| Age [mean (SD), years] | | 58.61 (10.14) | <1% | 58.28 (9.08) | 0% | 58.67 (9.36) | 0% |
| Education | Primary school and below | 11755 (66.59) | <1% | 4366 (60.82) | <1% | 7196 (66.59) | <1% |
|  | Middle/high/vocational school | 5469 (30.98) |  | 2637 (36.73) |  | 3405 (31.51) |  |
|  | Associate degree and above | 429 (2.43) |  | 176 (2.45) |  | 206 (1.91) |  |
| Marital status | Unmarried | 3505 (19.83) | <1% | 631 (8.78) | 0% | 1684 (15.58) | 0% |
|  | Married | 14170 (80.17) |  | 6558 (91.22) |  | 9126 (84.42) |  |
| Yearly income | Under average level | 9515 (65.65) | 7% | 3564 (61.75) | 7% | 5913 (64.9) | 5% |
|  | Average and above | 4979 (34.35) |  | 2208 (38.25) |  | 3198 (35.1) |  |
| Residence | Rural | 7715 (75.48) | 42% | 3066 (76.65) | 44% | 5185 (79.71) | 40% |
|  | Urban | 2506 (24.52) |  | 934 (23.35) |  | 1320 (20.29) |  |
| Smoke | Current smoker | 4862 (28.76) | 2% | 2231 (32.4) | 2% | 3403 (31.49) | <1% |
|  | Previous smoker | 1417 (8.38) |  | 597 (8.67) |  | 965 (8.93) |  |
|  | Never smoked | 10628 (62.86) |  | 4057 (58.93) |  | 6439 (59.58) |  |
| Drink | No | 11785 (67.14) | <1% | 4453 (62.24) | <1% | 7187 (66.48) | 0% |
|  | Yes | 5767 (32.86) |  | 2701 (37.76) |  | 3623 (33.52) |  |
| Length of sleep [mean (SD), hours] | | 6.37 (1.9) | 6% | 6.74 (1.64) | 8% | 6.44 (1.83) | <1% |
| Physical activity (SD) | | 52.83 (100.35) | 61% | 55.26 (101.78) | 61% | 59.4 (105.24) | 57% |
| Whole health status score (SD) | | 3.5 (1.02) | <1% | 3.28 (0.85) | <1% | 3.44 (1.00) | <1% |
| Childhood health status score (SD) | | 3.26 (1.07) | <1% | 3.34 (1.04) | <1% | 3.28 (1.07) | <1% |
| BMI | <18.5 kg / m^2^ | 947 (6.97) | 12% | 292 (5.11) | 21% | 623 (6.66) | 13% |
|  | 18.5-23.9 kg / m^2^ | 7179 (52.81) |  | 2991 (52.37) |  | 4973 (53.15) |  |
|  | 24-27.9 kg / m^2^ | 3938 (28.97) |  | 1754 (30.71) |  | 2727 (29.14) |  |
|  | ≥28 kg / m^2^ | 1530 (11.25) |  | 674 (11.8) |  | 1034 (11.05) |  |
| Number of difficulties in ADL (SD) | | 2.25 (2.82) | <1% | 1.28 (1.94) | 0% | 1.95 (2.44) | 0% |
| Number of difficulties in IADL (SD) | | 0.47 (1.11) | <1% | 0.19 (0.65) | 0% | 0.35 (0.92) | 0% |
| Number of non-communicable diseases (SD) | | 1.35 (1.4) | <1% | 1.06 (1.21) | 0% | 1.23 (1.28) | 0% |
| With major accidental injury | | 1603 (9.87) | 2% | 558 (8.39) | 2% | 1046 (9.68) | <1% |
| CRP | ≤3 mg/l | 9577 (82.11) | 34% | 4147 (83.34) | 31% | 6515 (82.72) | 27% |
|  | >3 mg/l | 2087 (17.89) |  | 829 (16.66) |  | 1361 (17.28) |  |

**Table S3** The Sensitivity Analysis of the Cohort Analysis for Baseline KOA and the Subsequent Risk of DS

|  | **Crude HR for incident DS** | |
| --- | --- | --- |
|  | **Participants Included in this research** | **Incident DS cohort with missing values for** **covariates** |
| Sample | 2582 | 7189 |
| With KOA at baseline | 2.05 (1.65-2.56) *** | 2.06 (1.80-2.36) *** |
| Without KOA at baseline | Reference | Reference |

**Table S4** The Sensitivity Analysis of the Cohort Analysis for Baseline DS and the Subsequent Risk of KOA

|  | **Crude HR for incident KOA** | |
| --- | --- | --- |
|  | **Participants Included in this research** | **Incident KOA cohort with missing values for covariates** |
| Sample | 4293 | 10810 |
| With DS at baseline | 2.52 (2.10-3.03) *** | 2.53 (2.25-2.84) *** |
| Without DS at baseline | Reference | Reference |

**Table S5** The Adjusted Model of Baseline KOA on the Subsequent Risk of DS (N=2582)

| **Variables** | | **HRs** | **95%CI** | | ***P*** |
| --- | --- | --- | --- | --- | --- |
| KOA | | 1.38 | 1.07 | 1.78 | 0.012 |
| Gender | Male | Reference | | | |
|  | Female | 1.51 | 1.22 | 1.89 | <0.001 |
| Age | | 0.98 | 0.97 | 0.99 | <0.001 |
| Education | Primary school and below | Reference | | | |
|  | Middle/high/vocational school | 0.77 | 0.64 | 0.92 | 0.003 |
|  | Associate degree and above | 0.98 | 0.51 | 1.86 | 0.95 |
| Marital status | Unmarried | Reference | | | |
|  | Married | 0.82 | 0.66 | 1.01 | 0.060 |
| Yearly income | Under average level | Reference | | | |
|  | Average and above | 0.81 | 0.70 | 0.94 | 0.006 |
| Residence | Rural | Reference | | | |
|  | Urban | 0.59 | 0.47 | 0.74 | <0.001 |
| Smoke | Never smoked | Reference | | | |
|  | Previous smoker | 1.03 | 0.75 | 1.40 | 0.87 |
|  | Current smoker | 1.17 | 0.95 | 1.44 | 0.14 |
| Drink | No | Reference | | | |
|  | Yes | 0.87 | 0.73 | 1.04 | 0.14 |
| Length of sleep | | 0.94 | 0.90 | 0.97 | 0.001 |
| Physical activity | | 1.00 | 1.00 | 1.00 | 0.18 |
| Whole health status score | | 0.76 | 0.69 | 0.84 | <0.001 |
| Childhood health status score | | 0.96 | 0.90 | 1.03 | 0.30 |
| BMI | <18.5 kg / m^2^ | Reference | | | |
|  | 18.5-23.9 kg / m^2^ | 1.12 | 0.80 | 1.58 | 0.50 |
|  | 24-27.9 kg / m^2^ | 1.03 | 0.72 | 1.47 | 0.88 |
|  | ≥28 kg / m^2^ | 0.89 | 0.60 | 1.33 | 0.58 |
| Number of difficulties in ADL | | 1.03 | 0.99 | 1.08 | 0.19 |
| Number of difficulties in IADL | | 0.97 | 0.86 | 1.08 | 0.55 |
| Number of non-communicable diseases | | 1.03 | 0.97 | 1.10 | 0.36 |
| With major accidental injury | | 0.90 | 0.69 | 1.18 | 0.46 |
| CRP | ≤3 mg/l | Reference | | | |
|  | >3 mg/l | 1.07 | 0.89 | 1.29 | 0.48 |

BMI = body mass index. ADL = activities of daily living. IADL = instrumental activities of daily living. CRP = C-reactive protein.

**Table S6** The Adjusted Model of Baseline DS on the Subsequent Risk of KOA (N=4293)

| **Variables** | | **HRs** | **95%CI** | | ***P*** |
| --- | --- | --- | --- | --- | --- |
| DS | | 1.51 | 1.23 | 1.84 | <0.001 |
| Gender | Male | Reference | | | |
|  | Female | 1.24 | 0.95 | 1.62 | 0.11 |
| Age | | 0.99 | 0.98 | 1.00 | 0.018 |
| Education | Primary school and below | Reference | | | |
|  | Middle/high/vocational school | 0.71 | 0.56 | 0.90 | 0.005 |
|  | Associate degree and above | 0.15 | 0.02 | 1.10 | 0.060 |
| Marital status | Unmarried | Reference | | | |
|  | Married | 0.99 | 0.80 | 1.22 | 0.90 |
| Yearly income | Under average level | Reference | | | |
|  | Average and above | 0.69 | 0.57 | 0.85 | <0.001 |
| Residence | Rural | Reference | | | |
|  | Urban | 1.07 | 0.82 | 1.39 | 0.61 |
| Smoke | Never smoked | Reference | | | |
|  | Previous smoker | 0.94 | 0.66 | 1.35 | 0.75 |
|  | Current smoker | 0.82 | 0.62 | 1.09 | 0.17 |
| Drink | No | Reference | | | |
|  | Yes | 0.98 | 0.78 | 1.23 | 0.85 |
| Length of sleep | | 0.96 | 0.92 | 1.01 | 0.095 |
| Physical activity | | 1.00 | 1.00 | 1.00 | 0.076 |
| Whole health status score | | 1.16 | 1.04 | 1.29 | 0.010 |
| Childhood health status score | | 0.98 | 0.90 | 1.06 | 0.56 |
| BMI | <18.5 kg / m^2^ | Reference | | | |
|  | 18.5-23.9 kg / m^2^ | 1.14 | 0.78 | 1.66 | 0.51 |
|  | 24-27.9 kg / m^2^ | 1.11 | 0.75 | 1.66 | 0.61 |
|  | ≥28 kg / m^2^ | 1.05 | 0.67 | 1.65 | 0.84 |
| Number of difficulties in ADL | | 1.09 | 1.05 | 1.14 | <0.001 |
| Number of difficulties in IADL | | 0.91 | 0.82 | 1.01 | 0.081 |
| Number of non-communicable diseases | | 1.39 | 1.31 | 1.48 | <0.001 |
| With major accidental injury | | 1.27 | 0.95 | 1.70 | 0.110 |
| CRP | ≤3 mg/l | Reference | | | |
|  | >3 mg/l | 1.16 | 0.93 | 1.45 | 0.20 |

BMI = body mass index. ADL = activities of daily living. IADL = instrumental activities of daily living. CRP = C-reactive protein.
